# Control system research in wave compensation based on particle swarm optimization

**DOI:** 10.1038/s41598-021-93973-4

**Published:** 2021-07-28

**Authors:** Gang Tang, Peng Lu, Xiong Hu, Shaoyang Men

**Affiliations:** 1 School of Logistics Engineering, Shanghai Maritime University, Shanghai, 201306 China; 2 School of Medical Information Engineering, Guangzhou University of Chinese Medicine, Guangzhou, 510006 China

**Keywords:** Electrical and electronic engineering, Energy infrastructure, Physical oceanography

## Abstract

For the offshore wave compensation control system, its controller setting will directly affect the platform's compensation effect. In order to study the wave compensation control system and optimization strategy, we build and simulate the wave compensation control model by using particle swarm optimization (PSO) to optimize the controller's control parameters and compare the results with other intelligent algorithms. Then we compare the response errors of the wave compensation platform under different PID controllers; and compare the particle swarm algorithm's response results and the genetic algorithm to the system controller optimization. The results show that the particle swarm algorithm is 63.94% lower than the genetic algorithm overshoot, and the peak time is 0.26 s lower, the adjustment time is 1.4 s lower than the genetic algorithm. It shows that the control effect of the wave compensation control system has a great relationship with the controller's parameter selection. Meanwhile, the particle swarm optimization algorithm's optimization can set the wave compensation PID control system, and it has the optimization effect of small overshoot and fast response time. This paper proposes the application of the particle swarm algorithm to the wave compensation system. It verifies the superiority of the method after application, and provides a new research reference for the subsequent research on the wave compensation control systems.

## Introduction

It is tough to use the equipment for transportation on the ocean, especially on some seas with more complicated sea conditions. When it is necessary to lift or carry some cargo, wave compensation technology is usually used to reduce the ship's attitude changes caused by waves. In the past few decades, wave compensation technology has been extensively developed. Wave compensation technology plays a crucial role in the exploration of marine resources. Various offshore cranes with wave compensation functions are widely used in marine-related operations^1–3^.

According to the wave compensation principle, it can be divided into passive wave compensation systems, active wave compensation systems, and compound wave compensation system. According to different compensation degrees of freedom, it can be divided into two degrees of freedom, three degrees of freedom, and six degrees of freedom wave compensation systems. Among them, the six-degree-of-freedom wave compensation system has received more and more attention because of its advantages in compensation effects. It can completely compensate for the relative movement between the two ships, and it not only can avoid collisions between the cargo and the ship being supplied, but also can accurately supply the cargo to the designated location^4–6^. Among them, the design and optimization of the control system play an extremely important role in the quality of wave compensation^7–12^.

In terms of controller design of wave compensation system, PID controller is the earliest proposed controller based on feedback, with simple structure and wide applicability, and it is widely used in compensation control systems. For the determination of the controller parameters, the classic tuning methods are mainly the Z-N method, etc., but these traditional methods lack flexibility and sometimes cause oscillation and large overshoot.

For the above reasons, many new optimization methods have been developed^13–17^, such as a fuzzy-based PID^18–22^, a neural network-based PID^23–25^. Among them, particle swarm optimization algorithms have the characteristics of simple operation, high accuracy, and fast convergence and are widely used^26–29^. Xia et al. have done a lot of research on the improvement and application of particle swarm algorithm^30–32^. The reason why particle swarm algorithm has gained so much attention is due to its unique advantages. The PID controller based on the particle swarm algorithm can optimize the factors of the controller to realize the intelligent adjustment of PID parameters. For the controllers of wave compensation platforms, optimization strategies based on intelligent algorithms have received more and more attention and development.

The paper is organized as follows. First, the principle and application of PSO are introduced in “Particle swarm algorithm process” section; then, in “PID controller of ship wave compensation system” section, the authors apply PID to the wave compensation system of a ship and explain the parameters therein. In “Wave compensation platform control system and simulation” section, the simulation is performed, and the effect of PID parameters on the wave compensation system is obtained. The PSO algorithm is compared with other intelligent algorithms, and the resulting image is obtained in “Intelligent algorithm optimization and comparison of wave compensation control system” section. Finally, In “Conclusion” section the full text is summarized, and the future outlook is presented.

## Particle swarm algorithm process

The PSO algorithm has made considerable progress. Ghooi et al.^33^ have combined the Kronecker summation method and a modified PSO search technique, gives an optimal or approximately optimal controller that is quite successful in comparison to classical techniques and even numerous modern control techniques. For the wave compensation platform under the PID controller, when the particle swarm algorithm is used to solve the optimization problem, the particles are the values of PID parameters Kp, Ki, Kd, and the optimal solution of the problem corresponds to the position of the particle in the search space. Each particle has speed and position and fitness value.

The flow of the particle swarm algorithm is as follows:

At first, initialize the particle swarm and initialize each particle randomly.

Determine the dimension N of the parameter, the initial position of the parameter of the PID controller of the wave compensation control system, the iterative velocity and position of the particle can be determined by the following formula:

1 $$v^{(j)} = w \cdot v_{0} + c_{1} \cdot r_{1} \cdot (p^{(j)} - x^{(j)} ) + c_{2} \cdot r_{2} \cdot (p_{g} - x^{(j)} )$$

2 $$x_{k + 1}^{(j + 1)} = x_{k}^{(j)} + v_{k}^{(j + 1)}$$

**Figure 1:**
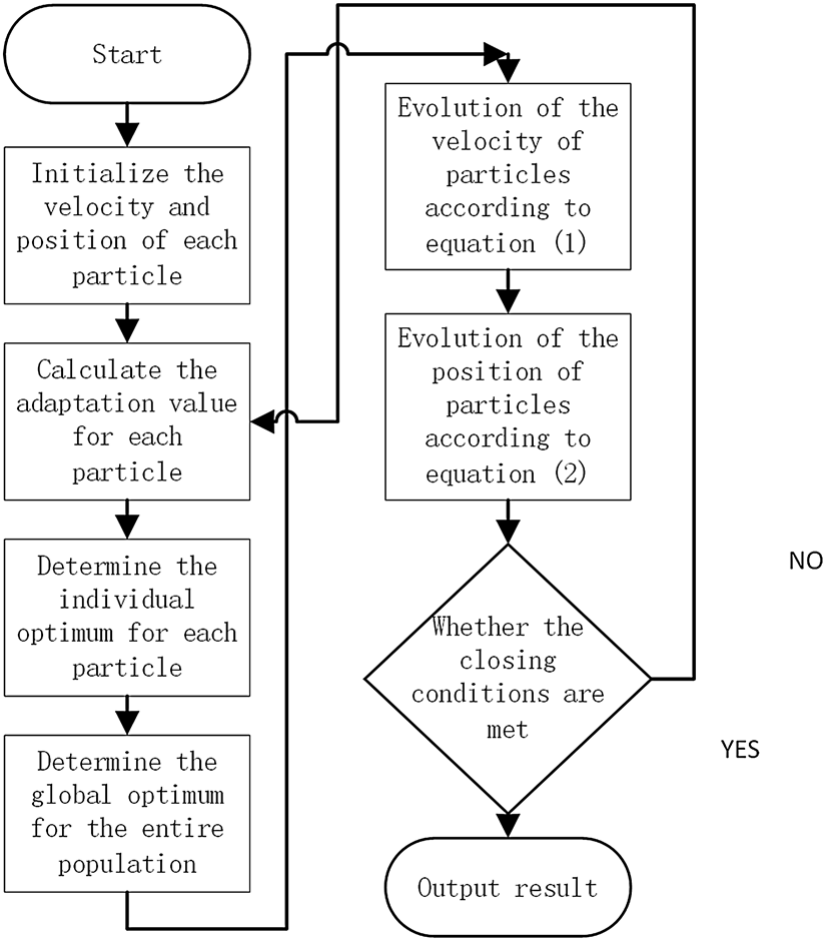
Flow chart of particle swarm algorithm.

The flow of particle swarm optimization algorithm is shown in Fig. 1.

## PID controller of ship wave compensation system

The history of PID control is very early. In current process control, many control loops have a PID structure, and many of them are based on PID control. The fundamental reason why it can be widely used is that it has a simple structure, clear logic, and it can adapt to many occasions and is robust. Nevertheless, for PID controllers, the use of different proportional coefficients, integral time constants, and derivative time constants has a great influence on the effect of the control system. Therefore, whether the optimal value of the parameter can be determined is very important to the control effect of the entire system. It can also be said that the optimization of PID parameters is the core of control system design.

**Figure 2:**
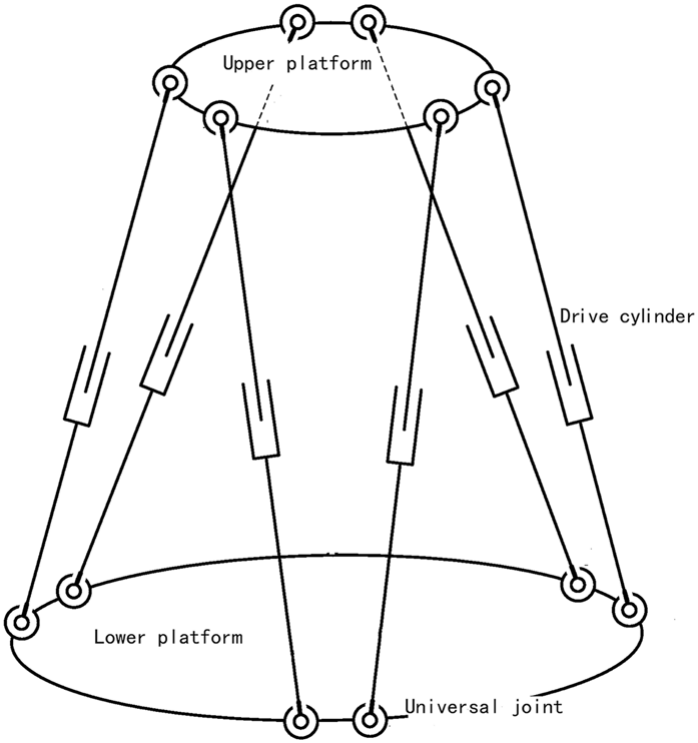
Schematic diagram of the structure of the wave compensation platform.

**Figure 3:**
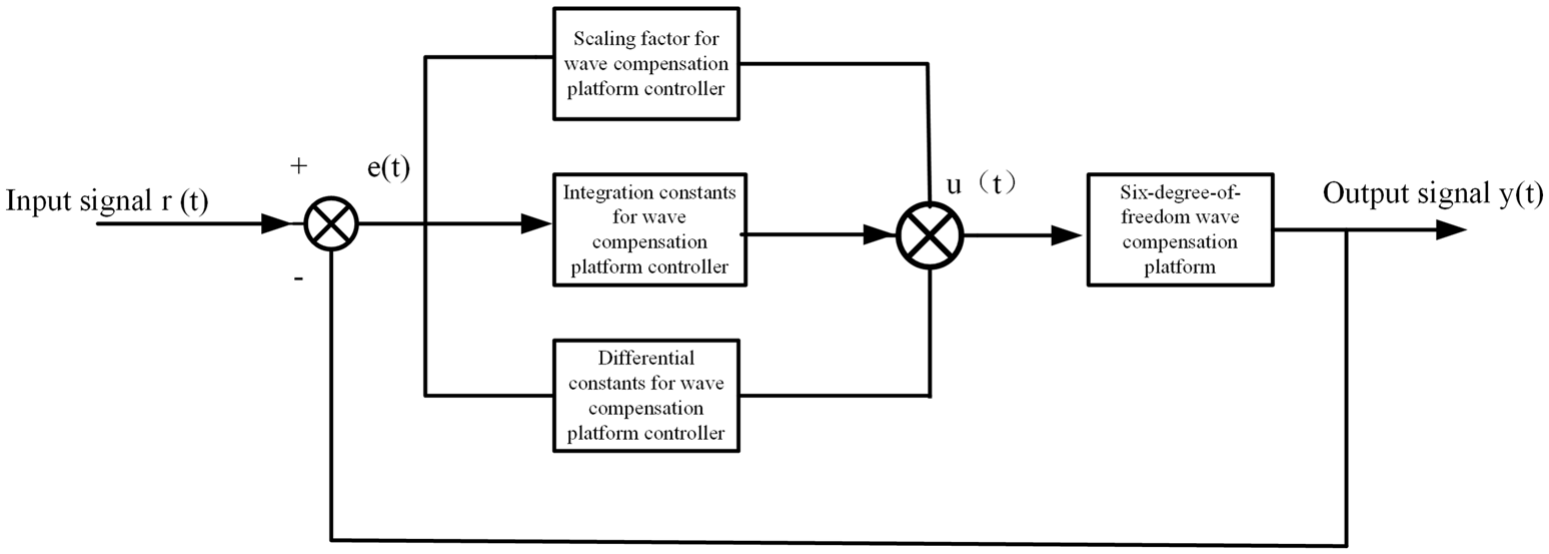
Diagram of PID control principle of ship wave compensation system.

Figure 2 is a schematic diagram of the ship's wave compensation platform structure, which is divided into an upper platform and a lower platform, connected by six driving cylinders. The lower platform is placed on the ground, and the upper computer controls the movement of the upper platform by controlling the elongation of each drive cylinder. Figure 3 is a block diagram of the PID control principle of the ship wave compensation system. The control system is composed of a PID controller and a controlled object. The controlled object is each drive cylinder of the designed wave compensation mechanism.

The PID control of the ship wave compensation system is a linear controller, which constitutes a control deviation according to the given value r(t) and the actual output value y(t):

3 $$e(t) = r(t) - y(t)$$

The PID control law of ship wave compensation system is:

4 $$u\left( t \right) = K_{p} \left( {e\left( t \right) + \frac{1}{{T_{i} }}\int_{0}^{t} {e\left( t \right)dt + \frac{{T_{d} de(t)}}{dt}} } \right)$$

The form of its transfer function:

5 $$G\left( s \right) = \frac{U\left( s \right)}{{E\left( s \right)}} = K_{p} + \frac{{K_{i} }}{s} + K_{d} s$$

Among them, $K_{p}$ is the proportional coefficient, $T_{i}$ is the integral time constant, and $T_{d}$ is the derivative time constant. The functions of each correction link in the PID controller of the ship wave compensation system are as follows:

1. The proportional link of the ship wave compensation system (proportional coefficient $K_{p}$): it reflects the error in a certain proportion. Once an error occurs, it is controlled immediately to reduce the error.
2. Integral link of ship wave compensation system (integration time constant $T_{i}$): to eliminate static error, the smaller the value of $T_{i}$, the stronger the integral effect; the larger the value of $T_{i}$, the weaker the integral effect.
3. Differential link of ship wave compensation system (differential time constant $T_{d}$): reflect the rate of change of deviation, add a parameter adjusted in advance to the compensation system, the smaller the value of $T_{d}$, the stronger the differential effect; the larger the value of $T_{d}$, the greater the differential effect weak.

The influence of PID parameters on controller performance is shown in Table 1.

**Table 1:** The influence of PID parameters on the performance of wave compensation controller.

Parameter	Value size	Effect	
		Responding speed	Overshoot
Scale factor $K_{p}$	Increase↑	Increase↑	
Integration time constant $T_{i}$	Increase↑		Reduce↓
Differential time constant $T_{d}$	Too large↑ Or too small↓	Reduce↓	Increase↑

## Wave compensation platform control system and simulation

**Figure 4:**
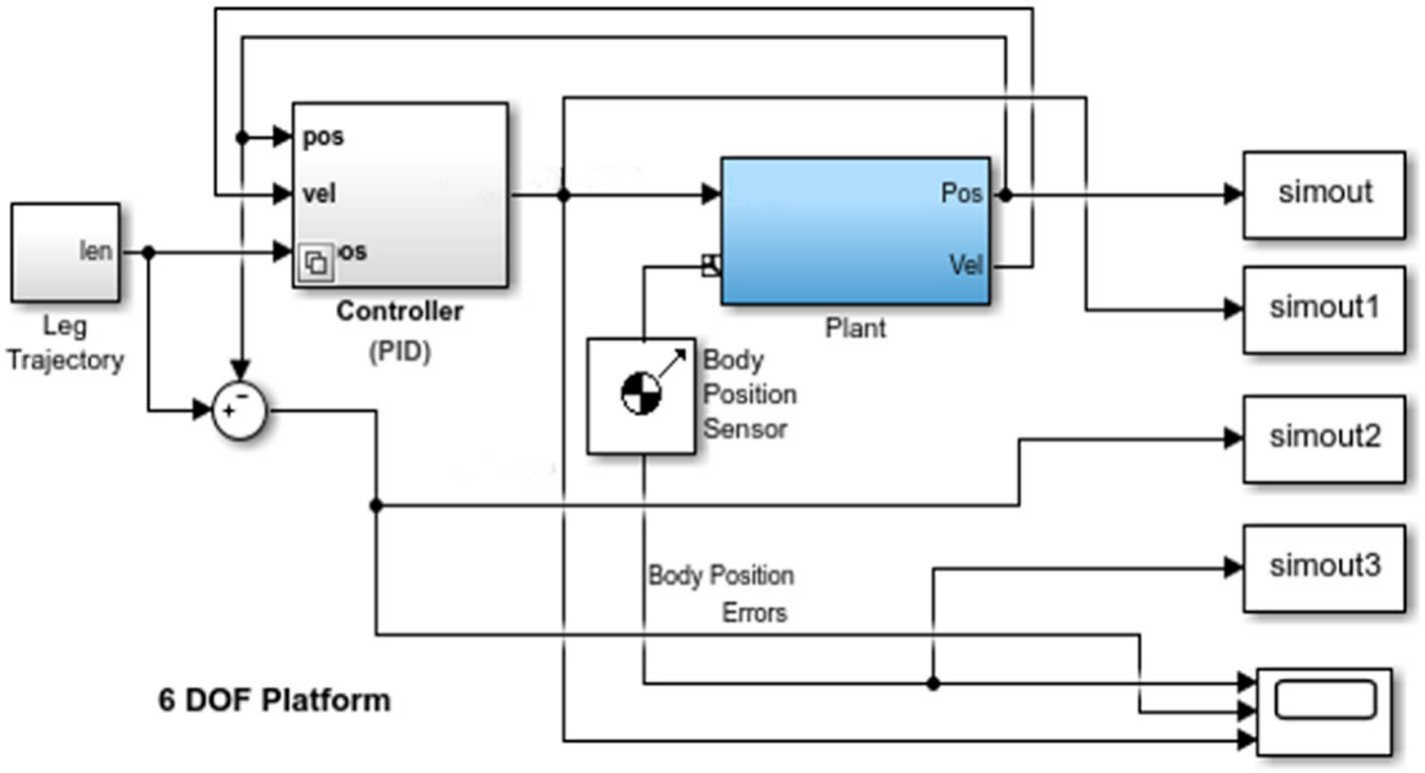
Diagram of the Simulink control system of the six-degree-of-freedom wave compensation platform.

**Figure 5:**
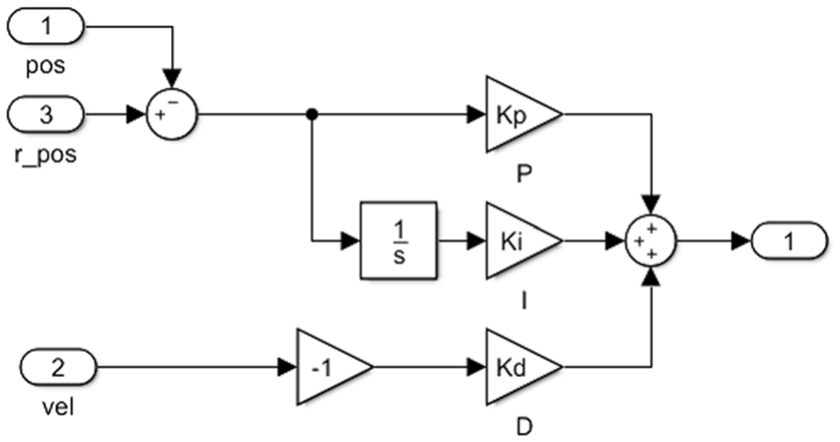
Diagram of PID controller Simulink of wave compensation platform.

**Figure 6:**
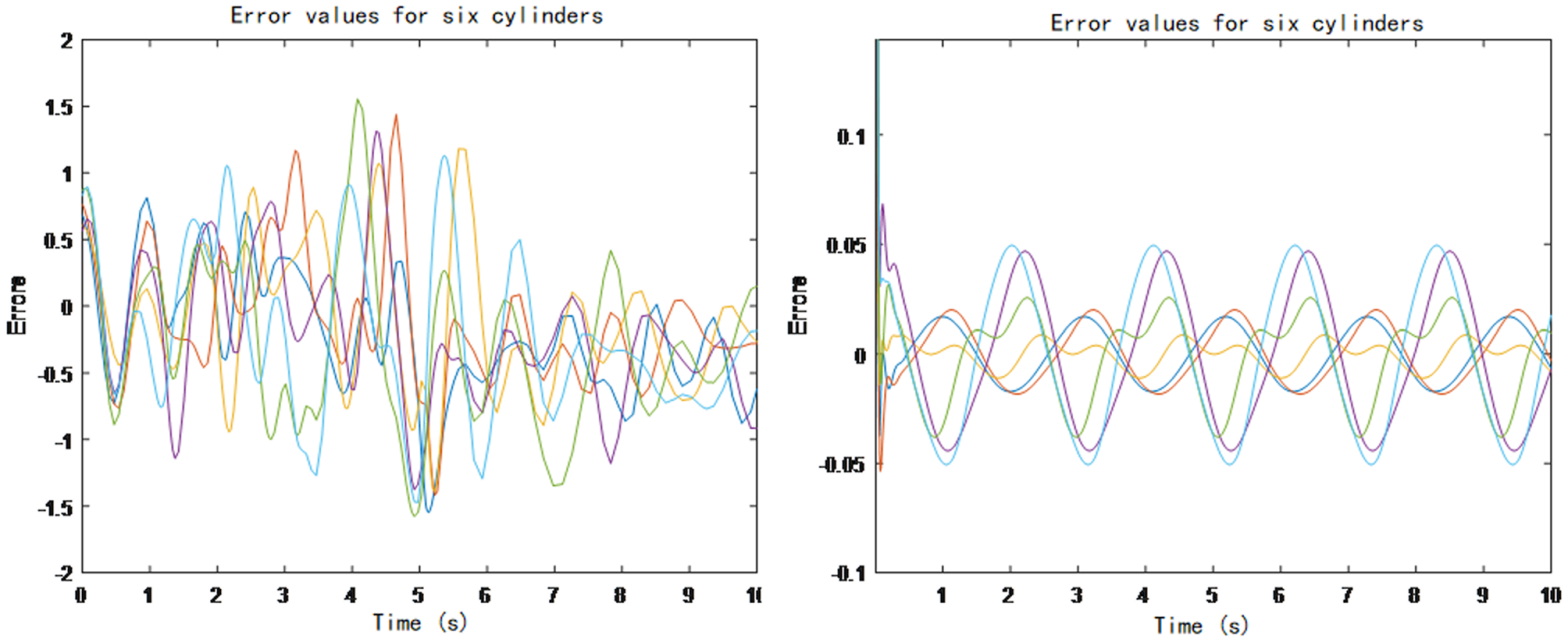
Errors obtained from the wave compensation platform test.

For the wave compensation platform, the control system can be shown in Fig. 4. Where the controller is a PID controller, as shown in Fig. 5. The upper platform is established as a dynamic coordinate system, and the lower platform is a static coordinate system. The position and posture of the upper platform are known, and the elongation of each cylinder is obtained by the inverse solution method. After that, the driving signal is input to the PID controller, and then into the platform system established by Sim Mechanics. The system outputs the position and sensor signals as Pos and Vel signals, which are fed back to the controller to form a closed loop control. The platform system Plant in the figure is established according to the structure of the wave compensation platform in Fig. 2. PID controller adjusts through feedback to reduce the existence of error. Because the PID parameters of different wave compensation platforms are different, we build a ship wave compensation system based on PID control. In order to show that the selection of its parameters will have an impact on the control effect, we select two groups of different parameters to show the difference. By adjusting the PID parameters, the error image of the model simulation is obtained. The specific values of the PID parameters used in the experiment are shown in Table 2 below, and the resulting image is shown in Fig. 6.

**Table 2:** PID parameters used in the test.

	Kp	Ki	Kd
Test 1	20,000	100	450
Test 2	2,000,000	10,000	45,000

Each curve in Fig. 6 represents the elongation error of a driving cylinder of the ship's wave compensation platform. When the PID parameters are 2,000,000, 10,000, 45,000, the error is obviously smaller than the error value when the parameters are 20,000, 100, 450. It can be seen that The parameters of PID controller are of great significance to the control accuracy of the wave compensation platform.

**Figure 7:**
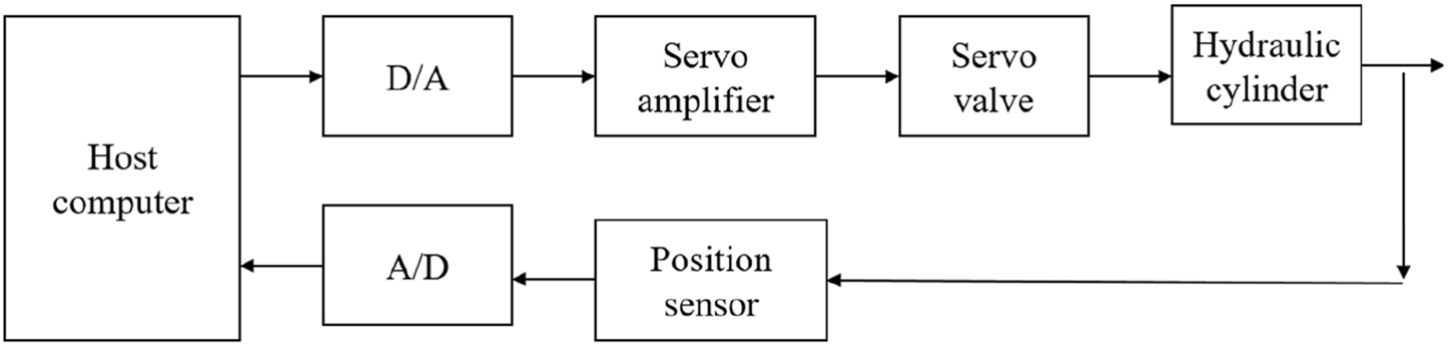
Schematic diagram of wave compensation control system.

As shown in Fig. 7, the six-degree-of-freedom wave compensation platform servo system. The control signal of the microcomputer system is converted into an analog voltage signal to control the servo valve through digital-to-analog conversion. The servo valve then converts the voltage signal into a signal that drives the drive cylinder. The position signal output of the drive cylinder passes the position sensor output voltage signal, which is converted into a digital signal through analog-to-digital conversion and transmitted to the microcomputer control system.

For the six-degree-of-freedom wave compensation platform, its single-channel drive transfer function can finally be transformed into a third-order transfer function. For the wave compensation control system, this paper uses a simplified transfer function to express:

6 $${\text{G}}(s) = \frac{s + 2}{{s^{{3}} + 7s^{{2}} + 4s + {1}}}$$

In addition, it is necessary to define a performance index as an objective function to evaluate the fitness of individual particles in the algorithm. The indicators for measuring a control system have three aspects: stability, accuracy, and speed. When adjusting PID parameters, we usually pay attention to the overshoot, rise time, adjustment time, peak time of the response curve. The combination of these parameters is used as an evaluation function. The smaller the evaluation function, the better the parameters obtained by simulation.

The error performance index selects ITAE (Integrated Time and Absolute Error) index:

7 $$J = \int_{0}^{t} {t\left| {e\left( t \right)} \right|} dt$$

The performance index of time multiplied by the integral of absolute value of error (ITAE) is a kind of control system performance evaluation index with good engineering practicality and selectivity. It is a performance index expressed by the integral expression of the function of the deviation between the expected output of the system and the actual output or the main feedback signal, and it is a measure of the excellent performance of the control system.

**Figure 8:**
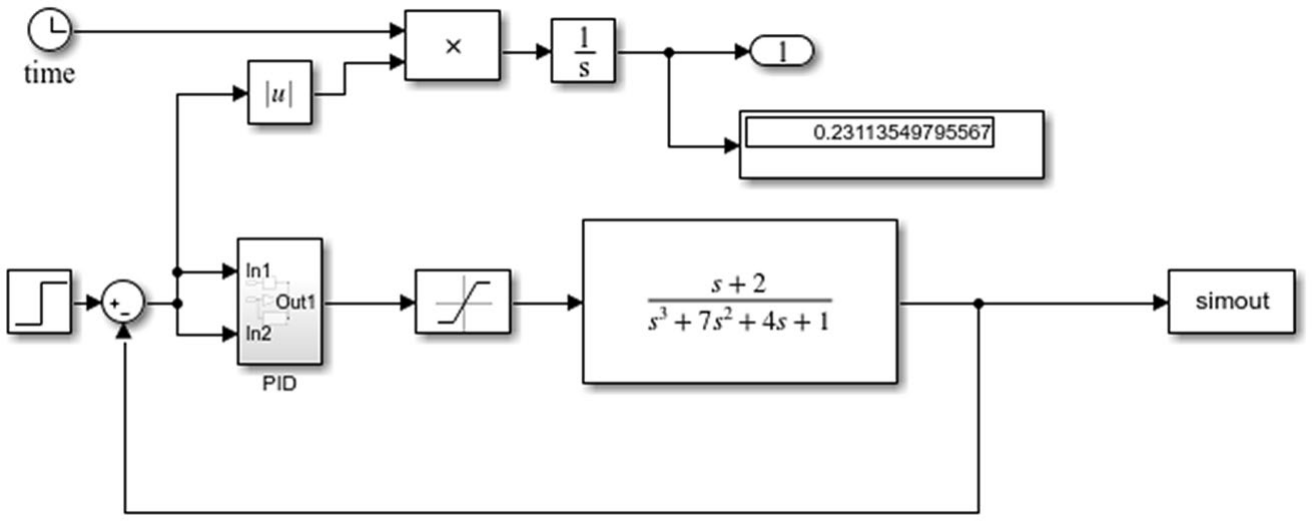
Diagram of wave compensation single channel Simulink control system.

Then we set up the control diagram in Simulink, as shown in Fig. 8.

## Intelligent algorithm optimization and comparison of wave compensation control system

The particle swarm algorithm is used to optimize the design of the PID controller, and the algorithm is used to adjust the gain of the controller. By taking the three parameters of the controller as the position of a particle in the algorithm, the parameter optimization problem of the controller can be easily transformed into a three-dimensional algorithm. Set up a control block diagram in the Simulink model, and connect the particle of the PID controller parameter to the performance index of the control system corresponding to the particle.

**Figure 9:**
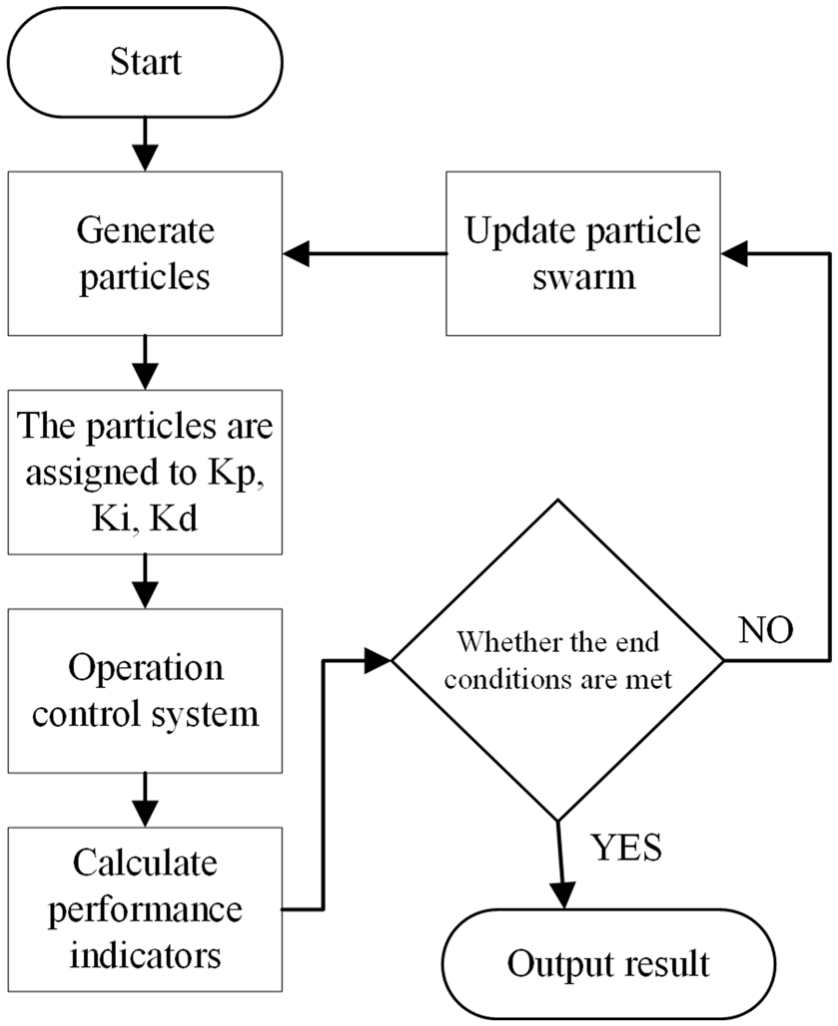
PID process of particle swarm optimization wave compensation control system.

The optimization process is shown in Fig. 9: the particle swarm generates a particle swarm (it can be an initialized particle swarm or an updated particle swarm), and the particles in the particle swarm are assigned to the parameters Kp, Ki, Kd of the PID controller in turn, And then run the Simulink model of the control system to obtain the performance index corresponding to the set of parameters, which is passed to the particle swarm as the fitness value of the particle, and finally, it is judged whether it can exit the algorithm.

Particle swarm algorithm related parameter settings: speed range: [− 1,1], PID parameter range: [0,300]. In addition, the inertia factor, acceleration constant, acceleration constant, maximum number of iterations, minimum fitness value, and population size of the particle swarm algorithm are shown in Table 3.

**Table 3:** Particle swarm algorithm parameter settings.

Parameter	Value	Parameter	Value	Parameter	Value
Inertia factor w	0.6	Acceleration constant c1	2	The maximum number of iterations M	100
Minimum fitness value	0.1	Acceleration constant c2	2	Population size D	100

**Figure 10:**
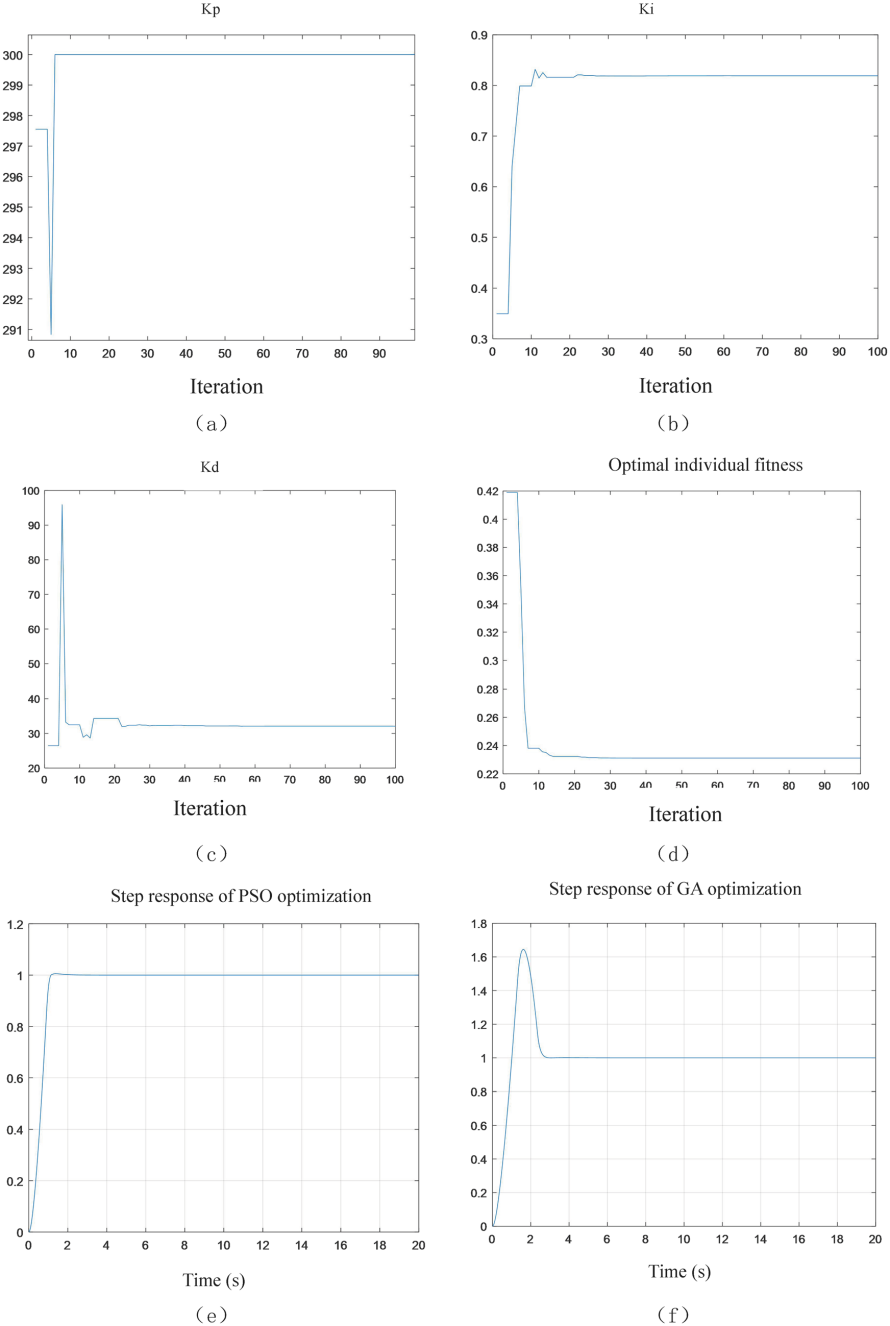
Optimization results of particle swarm optimization.

We can get the controller's proportional coefficient, integral time constant, derivative time constant and fitness value parameter iteration curve as shown in Fig. 10. As shown in Fig. 10a, after 10 iterations, the value of Kp reaches a stable value. As shown in Fig. 10b, after 12 iterations, the value of Ki reaches a stable value. As shown in Fig. 10c, after 23 iterations, Kd reaches a stable level. As shown in Fig. 10d, after 25 iterations, the fitness function converges.

The optimized parameters of particle swarm (PSO) are shown in Table 4, and the response curve is shown in Fig. 10e. At the same time, for the same model, genetic algorithm (GA) is used to optimize the parameters, and the numerical results are shown in Table 4, and the response curve is shown in Fig. 10f.

**Table 4:** Parameter optimization results.

Parameter	PSO optimization	GA optimization
ITAE	0.2311	1.2757
Kp	300	243.1764
Ki	0.8191	282.9052
Kd	32.1068	28.0034

**Figure 11:**
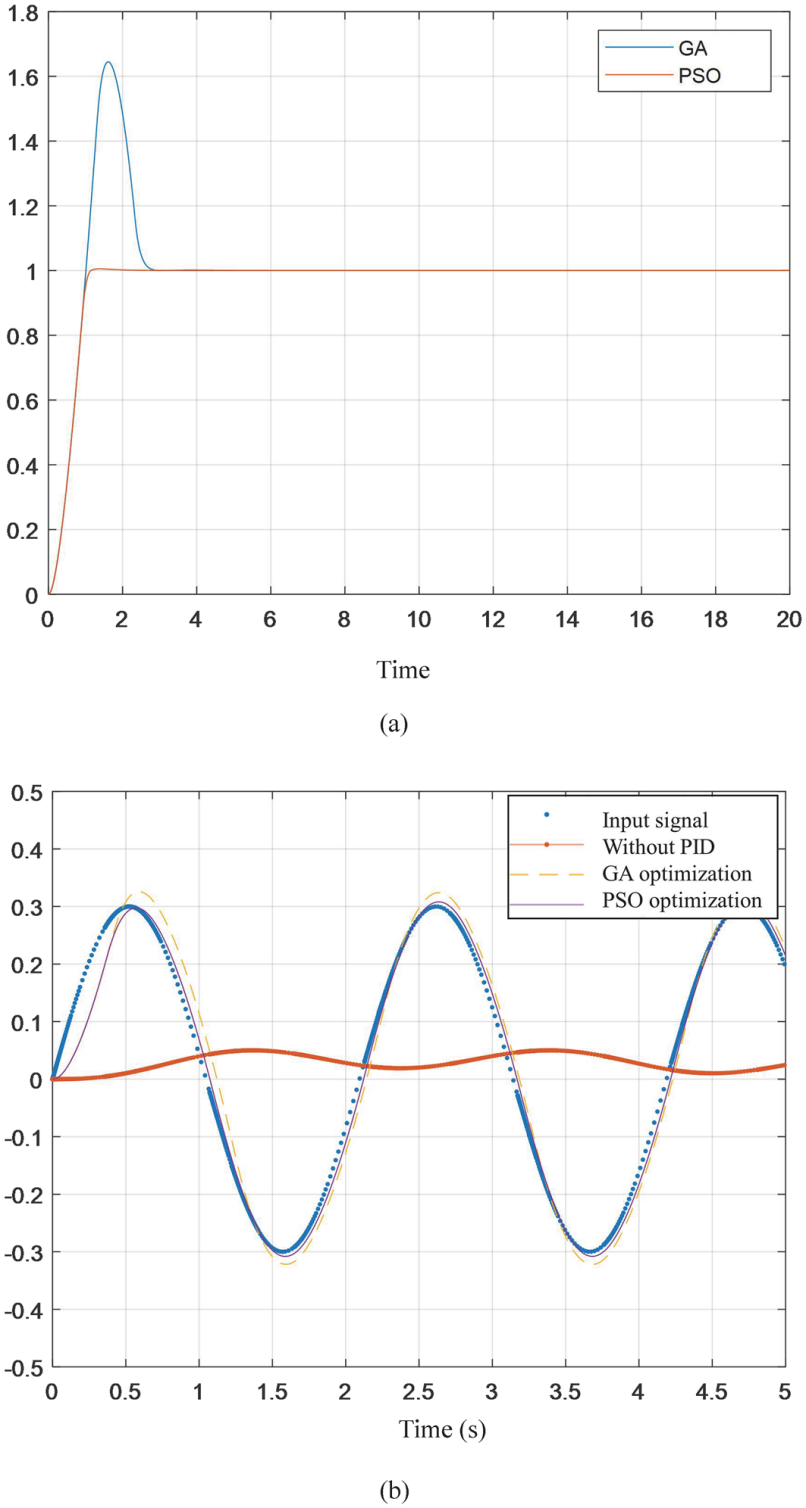
Response curve of the simulation system.

Bringing the obtained values into the system, we can get the wave compensation platform system response. The response curve comparison is shown in Fig. 11.

The analysis shows that for step input, the response overshoot obtained by the genetic algorithm is 64.49%, the peak time is 1.6194 s, and the adjustment time is 2.8 s; the step response obtained by the particle swarm algorithm, the overshoot is 0.55%, and the peak time is 1.3593 s, the adjustment time is 1.4 s. The overshoot particle swarm algorithm is 63.94% lower than the genetic algorithm, the peak time is 0.26 s lower, and the adjustment time is 1.4 s lower. The result is shown in Fig. 11a. The wave signal can be seen as a superposition of multiple sinusoidal signals. Using the same type of sinusoidal signal input in the third section of the simulation, the response curve shown in Fig. 11b is obtained. In summary, it can be seen that the system response obtained by particle swarm optimization, it has a smaller overshoot, a faster response speed, and a good control effect.

## Conclusion

This paper uses particle swarm optimization to optimize PID parameters and obtains the wave compensation control system's response curve. It is concluded that the control effect of the wave compensation control system is closely related to the controller's parameter selection. At the same time, the particle swarm optimization algorithm can tune the wave compensation PID control system. The effect of particle swarm optimization has a small overshoot and a fast response speed. Compared with other intelligent algorithms, it has obvious advantages. It has reference value for the optimization of wave compensation control systems.

PSO is an optimization algorithm based on iteration. The system is initialized to a group of random solutions, and then the optimal value is searched by iteration. The advantage of PSO is that it is simple and easy to implement, and there are not many parameters to adjust. But the PSO algorithm also has some shortcomings, such as the determination of some weight coefficients is not easy to choose. Therefore, other algorithms can be integrated into PSO algorithm in the future. Through appropriate adjustment, the advantages of each algorithm can be brought into play to improve the optimization ability of the algorithm. Mutation operation can be added to prevent premature algorithm, and improve the global optimization ability of PSO algorithm.
